# Phospholipase D6 activates Wnt/β-catenin signaling through mitochondrial metabolic reprogramming to promote tumorigenesis in colorectal cancer

**DOI:** 10.1038/s12276-025-01446-9

**Published:** 2025-04-21

**Authors:** Hyun Ji Lee, Seong Hun Lim, Hyesung Lee, Jung Min Han, Do Sik Min

**Affiliations:** 1 Department of Pharmacy, Yonsei University, Incheon, South Korea; 2 Yonsei Institute of Pharmaceutical Science, College of Pharmacy, Yonsei University, Incheon, South Korea

**Keywords:** Oncogenes, Cancer metabolism, Cell signalling

## Abstract

Phospholipase D6 (PLD6) is a critical enzyme involved in mitochondrial fusion with a key role in spermatogenesis. However, the role of PLD6 in cancer remains unknown. Notably, Wnt signaling, energy metabolism and mitochondrial function show complex interactions in colorectal cancer (CRC) progression. Here we found that PLD6 is highly expressed in CRC and positively correlated with poor prognosis. We present a novel function of PLD6 in activating Wnt/β-catenin signaling by enhancing mitochondrial metabolism. PLD6 depletion suppresses the oncogenic properties of CRC cells and impairs mitochondrial respiration, leading to reduced mitochondrial length, membrane potential, calcium levels and reactive oxygen species. PLD6 depletion also disrupts mitochondrial metabolic reprogramming by inhibiting the tricarboxylic acid cycle and mitochondrial oxidative phosphorylation, resulting in altered intracellular levels of citrate and acetyl-CoA—both key modulators of Wnt/β-catenin activation. PLD6-mediated acetyl-CoA production enhances β-catenin stability by promoting its acetylation via the acetyltransferases CREB-binding protein and P300/CREB-binding-protein-associated factor. Consequently, PLD6 ablation reduces cancer stem cell-associated gene expression downstream of Wnt/β-catenin signaling, suppressing stem-like traits and chemoresistance to 5-fluorouracil. Furthermore, PLD6 depletion attenuates CRC tumorigenesis in both subcutaneous and orthotopic tumor models. Overall, PLD6 acts as an oncogenic switch by promoting mitochondria-mediated retrograde signaling, thereby regulating Wnt signaling in CRC.

## PLD6 drives Wnt signaling and cancer progression in CRC

This study explores the role of a protein called phospholipase D6 (PLD6) in colorectal cancer (CRC). PLD6 is known to be involved in mitochondrial function, but its role in cancer was unclear. Researchers found that PLD6 is more active in CRC tissues compared with normal tissues, and higher levels of PLD6 are linked to worse outcomes for patients. Researchers used techniques such as CRISPR–Cas9 (a gene-editing tool) to modify PLD6 levels in cells and observed changes in cell behavior. They found that PLD6 promotes cancer cell growth, movement and survival by affecting mitochondrial metabolism and a signaling pathway called Wnt/β-catenin, which is important for cell growth and development. The study suggests that targeting PLD6 could be a potential strategy for treating CRC, as it may help reduce tumor growth and improve the effectiveness of chemotherapy.

This summary was initially drafted using artificial intelligence, then revised and fact-checked by the author.

## Introduction

Phospholipase D (PLD) catalyzes the hydrolysis of phospholipids, which are key components of cell membranes, to produce phosphatidic acid (PA), an intracellular lipid messenger that regulates critical cellular processes, such as proliferation, secretion, migration, vesicle trafficking, endocytosis and phagocytosis^1^. The mammalian PLD family comprises six isoforms, all of which contain HKD catalytic domains. The canonical isoforms PLD1 and PLD2 specifically hydrolyze phosphatidylcholine and possess two HKD domains that form a single active site. These isoforms also contain conserved tandem phox and pleckstrin homology domains, which are essential for their functionality^1^. In contrast, PLD3, PLD4, PLD5 and PLD6 are classified as noncanonical PLDs owing to the absence of phox and pleckstrin homology domains and inability to hydrolyze phosphatidylcholine into phosphatidic acid^2^. Notably, PLD3 and PLD4, despite lacking enzymatic PLD activity, function as 5′-exonucleases of DNA, enabling them to regulate inflammatory cytokine responses by degrading endosomal nucleic acids and modulating nucleic acid sensing. PLD5 is presumed to be catalytically inactive because of its insufficiently conserved catalytic domains; moreover, its functions remain largely uncharacterized^3^. PLD6, also known as mitochondrial PLD (MitoPLD), plays a dual role; it functions as an RNA endonuclease and regulates the biogenesis of PIWI-interacting RNA (piRNA), which is crucial for gene stability, modification and germ cell development^4–6^. The male mice lacking MitoPLD are sterile because of defects in piRNA biogenesis^5,7^. Furthermore, MitoPLD hydrolyzes cardiolipin on the mitochondrial surface to produce phosphatidic acid, which promotes mitochondrial fusion^8,9^. Unlike canonical PLDs, MitoPLD has a single HKD half-catalytic site and requires dimerization for lipase activity^8^. MitoPLD interacts with mitoguardin (MIGA) proteins, which stabilize it and promote its dimerization, thereby regulating mitochondrial phospholipid metabolism and enhancing mitochondrial fusion. However, MIGA proteins are not required for piRNA generation^10^. Mitochondrial dynamics play vital roles in development, stem cell differentiation and cellular fitness. Disrupted dynamics are associated with various diseases, and restoring balance in these processes has demonstrated potential to slow or prevent disease progression. Consequently, the proteins involved in mitochondrial fusion and fission have emerged as therapeutic targets^11–13^. Mitochondrial dynamics also regulate ATP production in response to energy demands. For example, cancer cells typically exhibit fragmented mitochondria and rely on glycolysis for rapid ATP production, despite its inefficiency compared with oxidative phosphorylation (OXPHOS)^12,14^. Beyond metabolism, mitochondrial dynamics influence processes such as apoptosis, calcium signaling and antiviral responses^15–17^. Dysregulation of these dynamics contributes to diseases, such as neurodegeneration, cancer and cardiac disorders, highlighting the therapeutic potential of targeting mitochondrial dynamics^18^. PLD1 and PLD2 are implicated in cancer by activating pathways central to tumor growth, cell cycle progression and angiogenesis. Aberrant expression of these isoforms has been observed in various cancers, and their inhibition has been demonstrated to reduce tumor growth and metastasis, positioning them as promising therapeutic targets^19,20^. However, the role of PLD6 in cancer remains unknown. Targeting mitochondrial metabolism has emerged as a critical therapeutic strategy for colorectal cancer (CRC) because CRC cells often exhibit a metabolic shift toward OXPHOS^21^. This reprogramming not only enables enhanced mitochondrial respiration for ATP production but also increases mitochondrial reactive oxygen species (ROS), which promote malignancy through DNA damage, oncogenic signaling and immune suppression^22,23^. Some CRC cell lines reveal higher respiratory capacity than that of other cancer types and inhibiting their reliance on mitochondrial respiration with electron transport chain inhibitors such as metformin results in antitumor effects^24^. PLD1 and PLD2 have been linked to CRC, and their inhibition suppresses cell growth and survival pathways such as Wnt and PI3K/Akt signaling^20,25^. Although mitochondria are critical for OXPHOS, they also generate metabolites that act as signaling molecules regulating tumor progression^26^. Mitochondrial OXPHOS is also associated with cancer stem cell maintenance in various cancers^27^. However, the mechanisms underlying mitochondrial regulation in CRC remain unclear. In this study, we investigate the functional role of mitochondrial metabolism in CRC tumor growth and initiation, focusing on the potential involvement of PLD6 in CRC progression.

## Materials and methods

### Cell culture

Caco-2 and HCT116 cells were cultured in Minimum Essential Medium (MEM, Welgene) and Roswell Park Memorial Institute (RPMI, Corning), respectively, supplemented with 10% fetal bovine serum (Corning) and 1% penicillin–streptomycin (Gibco) at 37 °C in 5% CO_2_.

### CRISPR–Cas9-KO infection

The CRISPR–Cas9 guide RNA was designed, synthesized (Supplementary Table 1) and transduced into HEK293T cells using packaging and envelope vectors. The viral particles were collected and used to infect Caco-2 cells with polybrene (Merck Millipore). After 48 h, puromycin selection was performed. All experiments were approved by the Yonsei University Institutional Biosafety Committee (IBC-A-202005-239-01).

### Clinical samples, TMA and immunohistochemistry

The colorectal tissues from 50 patients were obtained from the Pusan National University Hospital (institutional review board number 7001988-202209-BR-1681-01E). A tissue microarray (TMA) of 160 CRC samples was purchased from US Biomax. Immunohistochemistry (IHC) staining was performed according to standard procedures. The samples were deparaffinized at 65 °C for 1 h, rehydrated from xylene to 70% ethanol. Subsequently, the cells were subjected to antigen retrieval and blocking. The primary antibodies were incubated at 4 °C overnight. On day 2, the samples were incubated with an HRP-conjugated secondary antibody (Vector Laboratories) and ABC Reagent (Vector Laboratories) at room temperature for 30 min. Staining was performed using 3, 3 -diaminobenzidine (DAB, Vector Laboratories) and Mayer’s hematoxylin (Dako). For TMA, the IHC staining intensity was scored as negative (score 0), weakly positive (score 1), moderately positive (score 2) and strongly positive (score 3). The proportion of positive staining was categorized into five grades: <5% (score 0), 6–25% (score 1), 26–50% (score 2), 51–75% (score 3) and >75% (score 4). The two scores from each part were then multiplied to evaluate protein expression; a score of 0–3 was considered negative, and a score of 4–12 was considered positive. The primary antibodies applied to IHC staining are listed as follows: anti-PLD6 (HPA049345), anti-Ki67 (ab16667), anti-cleaved caspase-3 (Cell Signaling 9661), anti-E-cadherin (BD 560061), anti-claudin-5 (sc-374221), anti-occludin (Thermo 33-1500), anti-ZO-1 (Thermo 61-7300), anti-N-cadherin (BD 610921), anti-vimentin (Cell Signaling 5741), anti-fibronectin (BD 610078), anti-Ac-K49-β-catenin (Cell Signaling 9030), anti-β-catenin (sc-7199), anti-CD44 (proteintech 15675-1), anti-CD133 (MBS462020), anti-EpCAM (sc-66020) and anti-YAP (sc-101199).

### Western blot analysis

The proteins were extracted, separated using SDS–polyacrylamide gel electrophoresis and transferred to PVDF membranes (Merck Millipore). The membranes were blocked with 5% Bovine serum albumin (BSA) in Tris-buffered saline-Tween 20 (TBS-T) and incubated overnight with the following primary antibodies at 4 °C: anti-c-Myc (sc-789), anti-cyclin D1 (sc-753), anti-Flag (sc-166355), anti-PLD6 (MBS9204473), anti-β-actin (sc-47778), anti-E-cadherin (BD 560061), anti-occludin (Thermo 33-1500), anti-ZO-1 (Thermo 61-7300), anti-N-cadherin (BD 610921), anti-vimentin (Cell Signaling 5741), anti-P300/CBP-associated factor (PCAF) (sc-13124), anti-CBP (sc-7300), anti-HDAC6 (A301-342A); anti-SIRT1 (sc-15404), anti-CD44 (proteintech 15675-1), anti-CD133 (MBS462020), anti-EpCAM (sc-66020) and anti-cleaved caspase-3 (Cell Signaling 9661). The membranes were then incubated with secondary antibodies for 1 h. Finally, the membrane was detected using a chemiluminescent solution (Merck Millipore) and analyzed using ImageJ software.

### qRT–PCR

The RNA was extracted from cells using the easy-BLUE reagent (Intron Biotechnology, Korea) and reverse transcribed into complementary DNA using the PrimeScriptRT Master Mix kit (Takara, Japan) according to the manufacturer’s instructions. Using cDNA as a template, quantitative real-time PCR was performed using SYBR Green PCR Master Mix (Takara) on a CFX96 real-time PCR operating system (Bio-Rad). The relative expression levels were calculated using the 2^−ΔΔCq^ method (Supplementary Table 2).

### Viability assay

A total of 1 × 10^4^ cells were seeded in 96-well plates and incubated with growth medium for 48 h, after which the WST-1 assay was performed according to the manufacturer’s instructions (DoGenBio, Korea). The results of the WST-1 assay were measured at 450 nm using an Infinite M200 PRO microplate reader (Tecan, Männedorf, Switzerland). All viability assays were performed in triplicate.

### Colony formation assay

A total of 300 cells were seeded into six-well plates and incubated for 15 days. The cells were fixed with 4% paraformaldehyde for 15 min and stained with crystal violet (Sigma) for 30 min. The number of colonies was determined using ImageJ software as well as manually counted under a light microscope (colony formation efficiency = (clone number/incubated cell number) × 100).

### Wound healing assay

A total of 5 × 10^4^ cells were seeded in 12-well plates. A linear wound was scratched with a sterile micropipette tip, and closure was monitored after 24 h. The representative images were captured using bright-field microscopy.

### Transwell migration and invasion assay

For migration assays, the cells were seeded at a concentration of 5 × 10^4^ cells per milliliter with serum-free medium in the upper transwell chamber (Corning) and growth medium with 10% fetal bovine serum added to the lower chamber for 24 h. The migrated cells were fixed with 4% paraformaldehyde and stained with crystal violet. For cell invasion assays, the inserts were coated with Matrigel (BD Biosciences), and the migration assays were performed.

### Flow cytometry

The cell cycle analysis was performed by fluorescein isothiocyanate-bromodeoxyuridine (BrdU) and propidium iodide staining (BD Biosciences). The CD44^+^CD133^+^ populations were gated using specific antibodies (Miltenyi Biotec) and analyzed using FlowJo software.

### Mitochondria length and biomass

The mitochondrial length was assessed by staining with MitoTracker Red. The mitochondrial length was measured by tracing the mitochondria using the ImageJ software. The mitochondrial length was either binned into distinct categories (<1 µm, fragmented; 1–3 µm, intermediated; 3 µm, tubulated) or taken as an average.

### Mitochondria membrane potential and calcium level assay

The mitochondrial membrane potential (MMP) was detected using JC-1 (T3168, Invitrogen) according to the manufacturer’s instructions. The JC-1 dye is excited at a wavelength of 488 nm; its monomers emit at 527 nm while its aggregates emit at 590 nm. The calcium levels were detected using rhodamine (Rhod)-2AM (ab142780) via confocal imaging, according to the manufacturer’s instructions.

### Metabolism assays

Citrate, succinate, fumarate and acetyl-CoA levels were quantified using the respective kits (Enzychrom and Sigma-Aldrich). The ATP levels were measured using a luminescent ATP assay kit (Abcam). The Seahorse XF Cell Mito Stress Test Kit and XF Glycolytic Rate Assay Kit (Agilent Life Sciences) were used to measure mitochondrial oxygen consumption and glycolysis.

### Immunofluorescence

To detect intracellular proteins, immunofluorescence staining was performed using primary antibodies, and the corresponding fluorescence-tagged secondary antibodies. The following primary antibodies were used: anti-Ki67 (ab16667), anti-E-cadherin (BD 560061), anti-claudin-5 (sc-374221), anti-occludin (Thermo 33-1500), anti-ZO-1 (Thermo 61-7300), anti-N-cadherin (BD 610921), anti-vimentin (Cell Signaling 5741), anti-fibronectin (BD 610078), anti-Ac-K49-β-catenin (Cell Signaling 9030), anti-β-catenin (sc-7199), anti-CD44 (Proteintech 15675-1), anti-CD133 (MBS462020), anti-c-Myc (sc-789), anti-cyclin D1 (sc-753), anti-PCAF (sc-13124), anti-CBP (sc-7300), anti-HDAC6 (A301-342A) and anti-SIRT1 (sc-15404). The following secondary antibodies were used: Alexa Fluor 488 goat anti-mouse IgG (A-11032; 1:1,000 dilution), Alexa Fluor 488 goat anti-rabbit IgG (A-11008), Alexa Fluor 594 goat anti-mouse IgG (A-11005) and Alexa Fluor 594 goat anti-rabbit IgG (A-11012). The nuclei were stained with Hoechst 33342 (1 μg ml^−1^). The LSM710 system (Carl Zeiss) was used to acquire images and Zen 3.4 (Lite) (Carl Zeiss) software was used to analyze the images.

### ChIP

A total of 5 × 10^6^ cells were fixed with 1% paraformaldehyde, quenched with 125 mM glycine and washed with PBS. DNA shearing was performed using RIPA buffer (WSE-7420) and sonication, and the sheared chromatin was collected using centrifugation. Immunoprecipitation was performed using antibodies and protein A/G agarose beads (sc-2003). The antibodies used for immunoprecipitation were anti-β-catenin (sc-7199) and control IgG (sc-2025). The genomic DNA from chromatin immunoprecipitation (ChIP) was analyzed by real-time quantitative reverse transcription PCR (qRT–PCR) using the primers listed in Supplementary Table 3.

### Luciferase reporter assay

A total of 2 × 10^5^ cells were cultured in six-well plates and transfected with TOP/FOP Flash reporter and Renilla plasmids. At 24 h post transfection, the cells were treated with 150 ng ml^−1^ Wnt3a for 4 h, and the cell lysates were collected. The luciferase activity was detected using the Dual-Luciferase Reporter System (Promega). The TOP/FOP ratio was used to assess the T cell factor transcriptional activity.

### Sphere-forming assay

To investigate the self-renewal capacity of the cells, 1 × 10^4^ cells were seeded into 24-well ultralow attachment plates (Corning). The cells were cultured in media containing Dulbecco’s modified Eagle medium/F12K (1:1) (Corning and Welgene, respectively), hormone mix B27 (Invitrogen), 20 μg ml^−1^ human epidermal growth factor (Sigma), 20 ng ml^−1^ human basic fibroblast growth factor (Sigma) and penicillin–streptomycin, at 37 °C for 7 days.

### Tumor xenografts

Animal experiments were approved by the Institutional Biomedical Research Ethics Committee of Yonsei University. The cells (2 × 10^5^) were injected subcutaneously into 8-week-old male C57/BL6 mice (*n* = 5 per group). The tumor sizes were measured every two days using a digital caliper, and the tumor volumes were calculated using the following equation: *V* = (length (mm) × width^2^ (mm^2^))/2. The mice were sacrificed at 3 weeks post injection.

### Statistical analysis

All data are expressed as the mean ± standard deviation of at least three independent experiments. The analyses were performed using GraphPad Prism 7.0 (GraphPad Software), with *P* < 0.05. considered statistically significant. An unpaired Student’s *t*-test was used for comparing two groups. When more than two groups were compared, a two-way analysis of variance was performed, followed by Sidak’s multiple comparisons test. Correlation analysis for IHC was performed using the chi-square test.

## Results

### PLD6 is upregulated and associated with poor prognosis in CRC

**Fig. 1:**
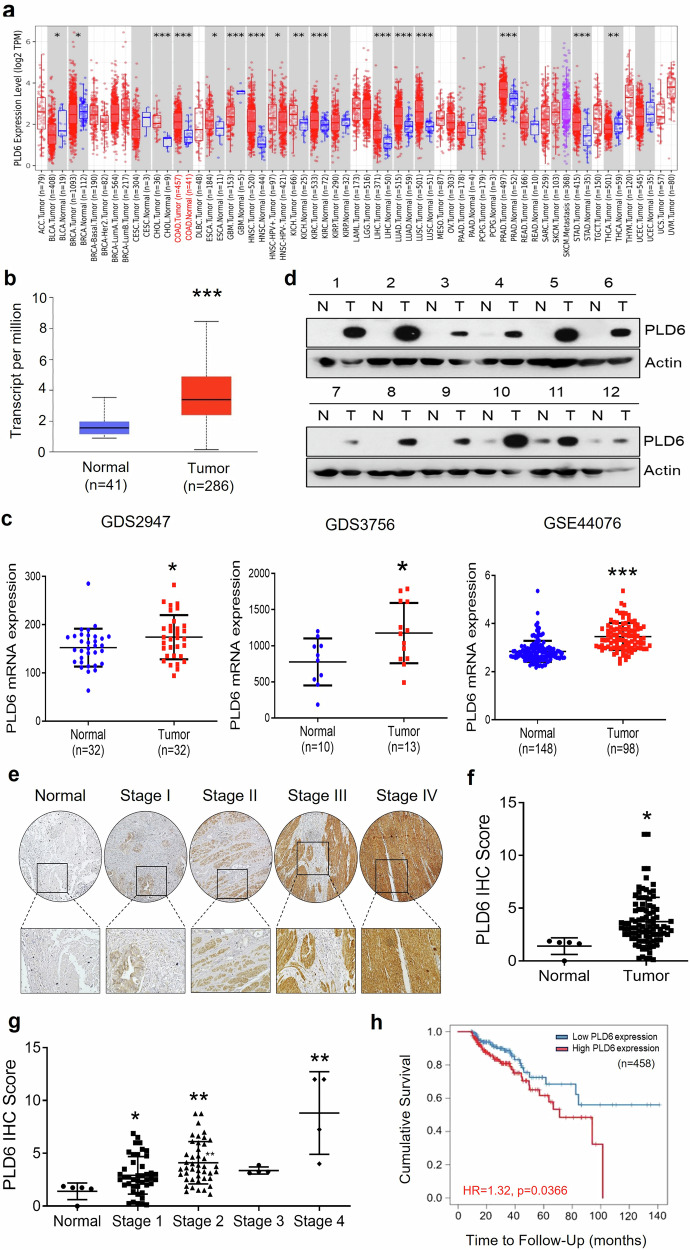
PLD6 is upregulated in CRC and is associated with poor prognosis. **a** The gene expression levels of PLD6 across various cancer subtypes in TCGA database were analyzed using TIMER2.0. The gray box plots indicate the cancer types with available data for matched normal tissues; the red and blue box plots represent tumor and normal samples, respectively. **b** A comparison of PLD6 expression between normal and tumor samples in CRC was performed using the UALCAN database. **c** The expression of PLD6 in patients with CRC compared with that in healthy controls was analyzed using datasets GDS2947, GDS3756 and GSE44076. **d** A western blot analysis of PLD6 expression in 12 paired CRC and adjacent normal tissue samples from patients. **e** An IHC analysis of PLD6 expression in CRC samples from patients, categorized by cancer stage. **f**, **g** The PLD6 expression levels in normal versus tumor tissues (**f**) and across different cancer stages (**g**). *P < 0.05, **P < 0.01, ***P < 0.001. **h**, The Kaplan–Meier survival curves of patients with CRC showing low and high PLD6 expression, as assessed using TIMER2.0.

To explore the role of PLD6 in cancer, we first analyzed its differential expression across various cancer types using TIMER2.0. The PLD6 expression was significantly elevated in various cancer tissues, including colorectal, breast, renal, lung, liver, prostate and stomach cancers (Fig. 1a). Notably, we chose to focus on CRC because the canonical PLD isoforms have been extensively studied in this cancer type^28–32^. We also utilized the UALCAN database and Gene Expression Omnibus (GEO) datasets to compare the mRNA levels in normal and tumor tissues (Fig. 1b, c). PLD6 expression was markedly upregulated in CRC tissues compared with that in normal tissues in both the UALCAN database and GEO datasets. Using western blot analysis, we confirmed that PLD6 protein levels were significantly elevated in CRC tissues relative to their normal counterparts (Fig. 1d). In addition, we examined PLD6 protein levels in surgically resected CRC samples from 136 patients using IHC. The relationship between PLD6 expression and cancer stage is summarized in Supplementary Table 4. The proportion of PLD6-positive samples increased significantly with advancing cancer stage (*p* < 0.001) (Fig. 1e). An IHC scoring was used to validate PLD6 upregulation in CRC tissues compared with that in normal tissues (Fig. 1f) and to confirm a positive correlation between PLD6 expression and pathological stage (Fig. 1g and Supplementary Table 4). Finally, using data from The Cancer Genome Atlas (TCGA), high PLD6 expression in patients with CRC was demonstrated to be strongly associated with poor prognosis (Fig. 1h). Collectively, these findings indicate that PLD6 is upregulated in CRC and is correlated with unfavorable clinical outcomes.

### PLD6 enhances the oncogenic properties of CRC cells

**Fig. 2:**
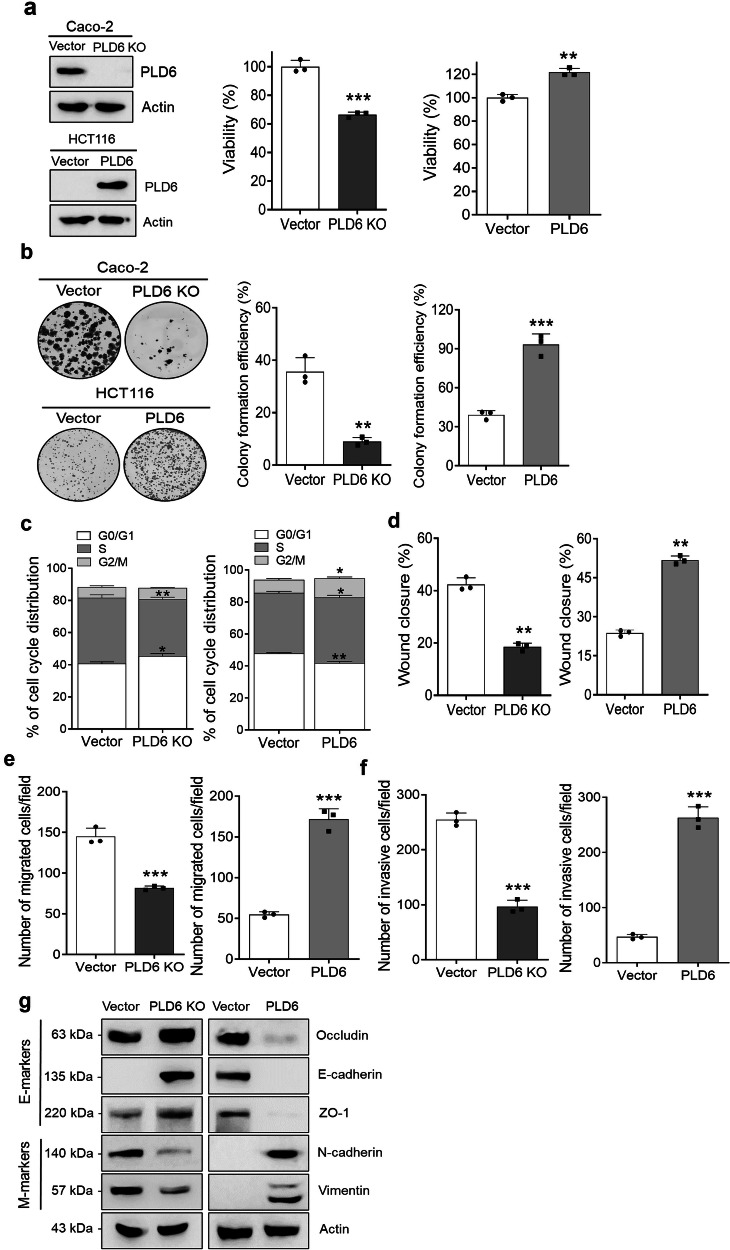
PLD6 enhances the oncogenic properties of CRC cells. **a** The cell viability was assessed using the WST-1 assay in PLD6-KO Caco-2 cells and PLD6-overexpressing HCT116 cells. **b** The colony-forming assays were performed with Caco-2 and HCT116 cells after manipulating PLD6 expression. **c** The cell proliferation was analyzed using a two-parameter cell cycle assay, incorporating flow cytometry to evaluate DNA content and BrdU incorporation. **d**, **e** The cell migration was evaluated using wound healing and transwell migration assays. For the transwell assay, 1 × 10^4^ Caco-2 cells and 5 × 10^4^ HCT116 cells were seeded into the upper chamber of the transwell. **f** The invasive potential was assessed using transwell chambers coated with Matrigel, with the same number of cells seeded as in the migration assay. **g** The changes in EMT markers were detected using western blot analysis of Caco-2 and HCT116 cells. The results represent at least three independent experiments. Statistical significance: *P < 0.05, **P < 0.01, ***P < 0.001.

To investigate the role of PLD6 in CRC cells, we generated PLD6-knockout (KO) Caco-2 cells, which naturally express endogenous PLD6, and PLD6-overexpressing HCT116 cells (Fig. 2a). Loss of PLD6 expression significantly reduced the viability of Caco-2 cells, whereas ectopic PLD6 expression enhanced the viability of HCT116 cells (Fig. 2a). The results of clonogenic assays, a key method for assessing cell survival and colony-forming ability, were consistent with those of the viability assays, further confirming that PLD6 promotes cell proliferation (Fig. 2b). To examine the role of PLD6 in regulating cell proliferation, we performed flow cytometric analysis of BrdU incorporation and DNA content. PLD6 deficiency reduced the proportion of cells in the S phase, while increasing the population in the G0/G1 phase (Fig. 2c and Supplementary Fig. 1a), suggesting a block in cell cycle progression. Conversely, PLD6 overexpression increased the S-phase population. These results indicate that PLD6 enhances cell proliferation by facilitating S-phase progression. Next, we evaluated the impact of PLD6 on cell migration using wound healing and transwell assays. The wound closure rates revealed that PLD6 significantly enhanced the migratory capacity of CRC cells (Fig. 2d and Supplementary Fig. 1b). Similarly, PLD6-overexpressing cells exhibited a significant increase in migration, whereas PLD6-KO cells displayed reduced migration (Fig. 2e and Supplementary Fig. 1c). The invasive potential of CRC cells followed a pattern consistent with the effect of PLD6 on cell migration (Fig. 2f and Supplementary Fig. 1d). Epithelial–mesenchymal transition (EMT), a key driver of cancer cell migration and invasion, is associated with increased malignancy. PLD6 deficiency elevated the expression of epithelial markers (E-markers) such as occludin, E-cadherin and ZO-1, while reducing the expression of mesenchymal markers (M-markers), including N-cadherin and vimentin (Fig. 2). In contrast, PLD6 overexpression promoted EMT by decreasing the E-marker levels and increasing the M-marker levels (Fig. 2g). Collectively, these findings suggest that PLD6 enhances the oncogenic potential of CRC cells by promoting their proliferation, migration, invasion and EMT.

### PLD6 regulates mitochondrial dynamics and membrane potential through its catalytic activity

**Fig. 3:**
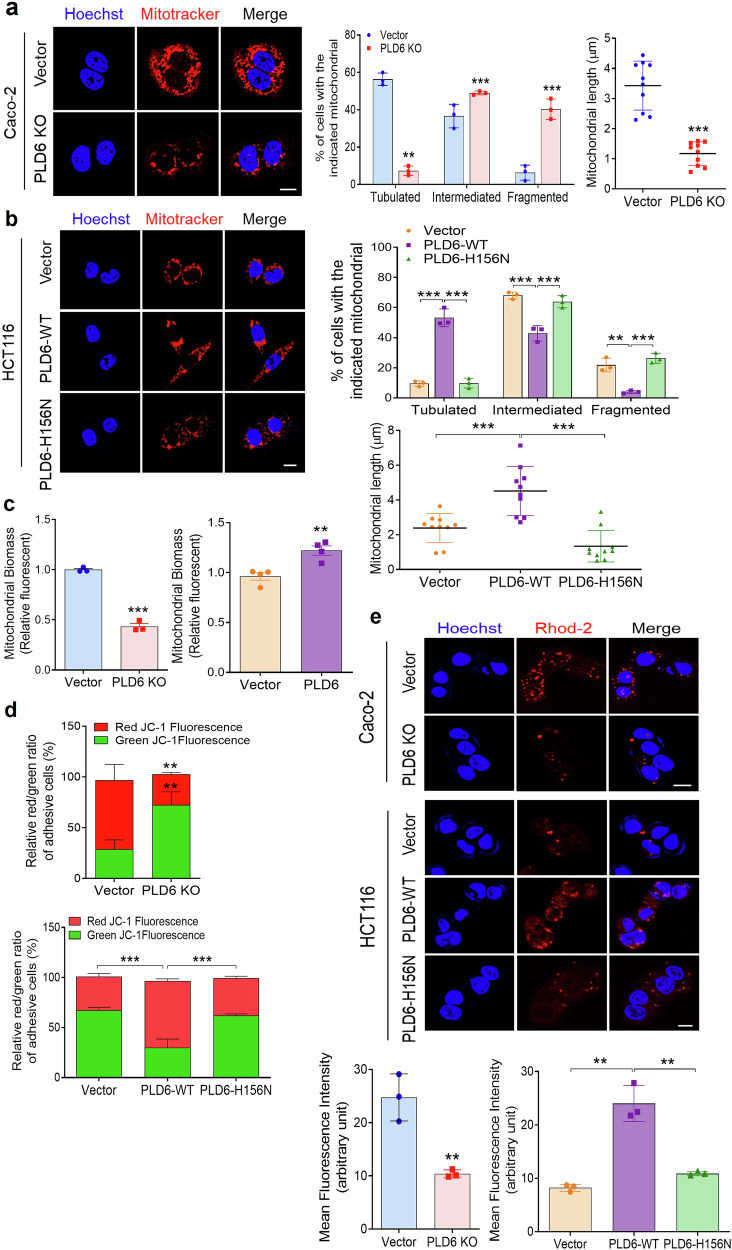
PLD6 activity is required for regulating mitochondrial dynamics and membrane potential. **a** The representative confocal microscopy images depict the effect of PLD6 KO on mitochondrial morphology in Caco-2 cells. The mitochondrial length and fragmentation were quantified using ImageJ. **b** The mitochondrial morphology in the indicated HCT116 cells was visualized, with mitochondrial length and fragmentation quantified using ImageJ. **c** The mitochondrial biomass in vector control and PLD6-altered cells was measured using MitoTracker Red and analyzed via flow cytometry. **d** The MMP in the indicated CRC cells was assessed using JC-1 dye. **e** The mitochondrial calcium levels in the indicated cells were measured using Rhod-2AM dye. The representative images were obtained from at least three fields. The results represent at least three independent experiments. Scale bar, 100 μm. Statistical significance: *P < 0.05, **P < 0.01, ***P < 0.001.

Considering that PLD6 is essential for mitochondrial fusion, we hypothesized that it may significantly regulate mitochondrial function and cancer metabolism. To explore this, we first investigated the effect of PLD6 on mitochondrial dynamics in CRC cells. Immunofluorescence analysis using MitoTracker Red, a dye that labels and tracks mitochondria, revealed that PLD6-deficient Caco-2 cells exhibited more fragmented mitochondria than those in control cells (Fig. 3a). In contrast, PLD6-overexpressing HCT116 cells displayed a significantly higher proportion of tubular mitochondria (Fig. 3b), confirming that PLD6 promotes mitochondrial fusion in CRC cells. Next, we assessed whether the catalytic activity of PLD6 influences mitochondrial dynamics. The PLD6-H156N mutant, a catalytically inactive form, resulted in reduced mitochondrial length compared with that with the PLD6 wild type (WT), suggesting that the catalytic activity of PLD6 is essential for promoting mitochondrial fusion (Fig. 3b). Moreover, PLD6-deficient CRC cells showed reduced mitochondrial biomass, as determined by MitoTracker Red staining and flow cytometry. In contrast, PLD6 overexpression resulted in an increased mitochondrial biomass (Fig. 3c). To further investigate the impact of PLD6 on MMP (Δψm), we employed the JC-1 dye, which assesses changes in MMP based on the formation of JC-1 aggregates (high MMP) that emit red fluorescence or monomers (low MMP) that emit green fluorescence. PLD6 ablation significantly decreased the red/green fluorescence ratio, indicating mitochondrial depolarization. Conversely, ectopic PLD6 expression led to the hyperpolarization of MMP, as evidenced by increased red fluorescence from JC-1 aggregates. The cells expressing the PLD6-H156N mutant exhibited reduced MMP, similar to that observed in PLD6-deficient cells (Fig. 3d and Supplementary Fig. 2a). As mitochondrial calcium uptake and release are closely linked to MMP, we assessed mitochondrial calcium levels using Rhod-2AM, a dye specific for mitochondrial calcium. PLD6 KO reduced mitochondrial calcium levels compared with those in the control cells, whereas PLD6 overexpression significantly increased the mitochondrial calcium levels (Fig. 3e). The PLD6-H156N mutant showed reduced mitochondrial calcium levels, indicating that the catalytic activity of is required to maintain proper mitochondrial calcium levels. Collectively, these results suggest that PLD6 activity is essential for regulating mitochondrial dynamics and membrane potential.

### PLD6 plays an oncogenic role in CRC cells by enhancing mitochondrial metabolism and OXPHOS

**Fig. 4:**
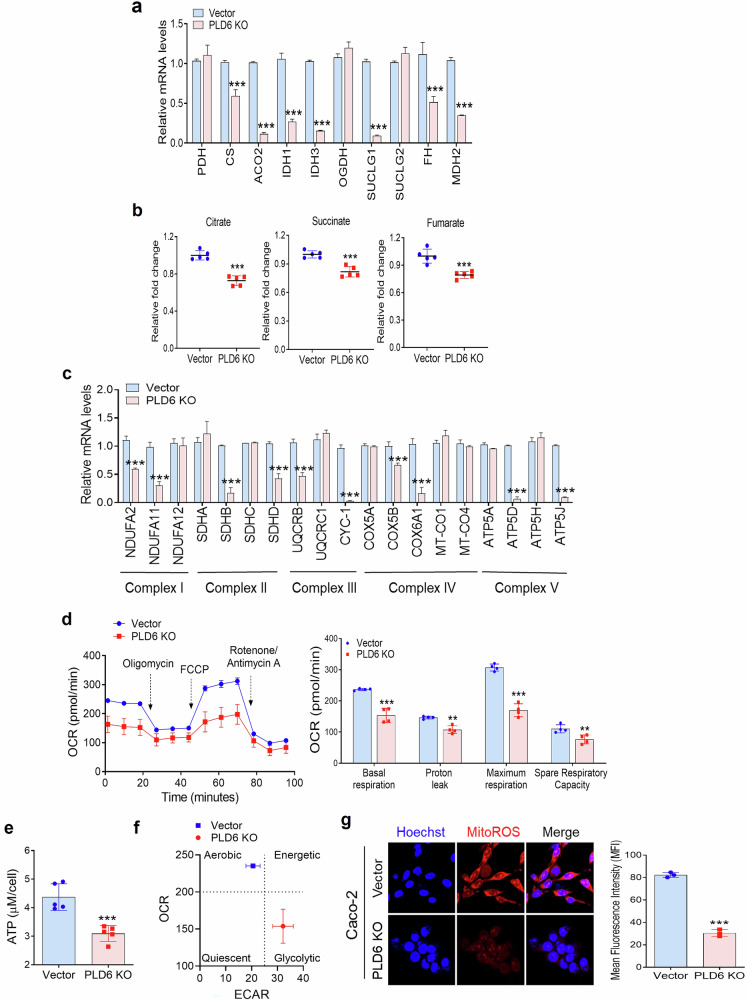
PLD6 plays an oncogenic role in CRC cells by enhancing mitochondrial metabolism and OXPHOS. **a** The expression levels of TCA cycle genes were measured in the indicated cells using qRT–PCR. **b** The TCA cycle metabolites were quantified in the indicated CRC cells. **c** The expression levels of the electron transport chain (ETC) complex genes were analyzed using qRT–PCR. **d** The oxygen consumption rate (OCR) was assessed using the Seahorse Extracellular Flux Analyzer. **e** The ATP production was measured using the Promega GloMax assay. **f** The energy phenotype profile, represented as a scatter plot, combines the OCR and extracellular acidification rate (ECAR) analysis. **g** The mitochondrial ROS levels were visualized using confocal microscopy and quantified. The representative images were obtained from at least three fields. The results represent at least three independent experiments. Scale bar, 100 μm. Statistical significance: *P < 0.05, **P < 0.01, ***P < 0.001.

We investigated the mechanism by which PLD6 regulates mitochondrial metabolic processes, including the tricarboxylic acid (TCA) cycle and OXPHOS. PLD6 KO in Caco-2 cells significantly reduced the transcriptional levels of seven key TCA cycle genes: citrate synthase (CS), aconitase 2 (ACO2), isocitrate dehydrogenase 1 (IDH1), isocitrate dehydrogenase 3 (IDH3), succinate-CoA ligase GDP-forming subunit beta (SUCLG1), fumarate hydratase (FH) and malate dehydrogenase (MDH) (Fig. 4a). We also assessed the TCA cycle metabolites and observed that PLD6 deficiency reduced the levels of citrate, succinate and fumarate, whereas PLD6 overexpression significantly increased their levels (Fig. 4b and Supplementary Fig. 2b). These metabolic alterations were dependent on PLD6 catalytic activity, as the PLD6-H156N mutant exhibited reduced TCA cycle metabolite levels. Moreover, PLD6 deficiency impaired mitochondrial OXPHOS by reducing the expression of genes associated with the electron transport chain, with 10 of 19 genes significantly downregulated in PLD6-deficient cells (Fig. 4c). Using the Seahorse Extracellular Flux Analyzer, we confirmed that PLD6 KO decreased oxygen consumption rates for both basal and maximal respiration, whereas PLD6 overexpression enhanced the oxygen consumption rates (Fig. 4d and Supplementary Fig. 2c). This result highlights the pivotal role of PLD6 in regulating mitochondrial respiration. Considering the link between mitochondrial respiration and ATP production, we found that PLD6 KO reduced ATP levels, whereas PLD6 overexpression increased them (Fig. 4e and Supplementary Fig. 2d). Importantly, the PLD6-H156N mutant could not restore ATP production to levels comparable with those in the PLD6-WT. Most CRC cells maintained an aerobic metabolic state^33^; however, PLD6 inhibition shifted the cells to a glycolytic state (Fig. 4f and Supplementary Fig. 2e). PLD6 overexpression caused more pronounced changes in the aerobic state than in the controls, mediated by its catalytic activity (Supplementary Fig. 2e). Altered levels of ROS in the cancer cell microenvironment have been linked to recurrence or relapse^34^. As mitochondrial respiration typically elevates mitochondrial ROS, we analyzed the ROS levels in PLD6-altered cells. Overexpression of PLD6-WT significantly elevated the mitochondrial ROS levels, whereas the PLD6-H156N mutant showed lower ROS levels (Supplementary Fig. 2f). PLD6 KO reduced mitochondrial ROS production compared with that in the control cells (Fig. 4g). Next, we examined whether mitochondrial metabolism mediated by PLD6 influences cancerous phenotypes. Mitochondrial respiration inhibitors such as metformin, rotenone and oligomycin significantly suppressed the viability of HCT116 cells (Supplementary Fig. 3a). However, PLD6-WT overexpression reduced the effects of these inhibitors, indicating that PLD6-enhanced mitochondrial metabolism supports CRC cell survival. Moreover, metformin significantly reduced the clonogenic and invasive capacities of HCT116 cells (Supplementary Fig. 3b, c). PLD6-WT overexpression but not the PLD6-H156N mutant restored the capacities suppressed by metformin (Supplementary Fig. 3b, c), indicating that PLD6-enhanced mitochondrial metabolism plays a significant oncogenic role in CRC cells.

### PLD6 upregulates Wnt/β-catenin signaling pathways

**Fig. 5:**
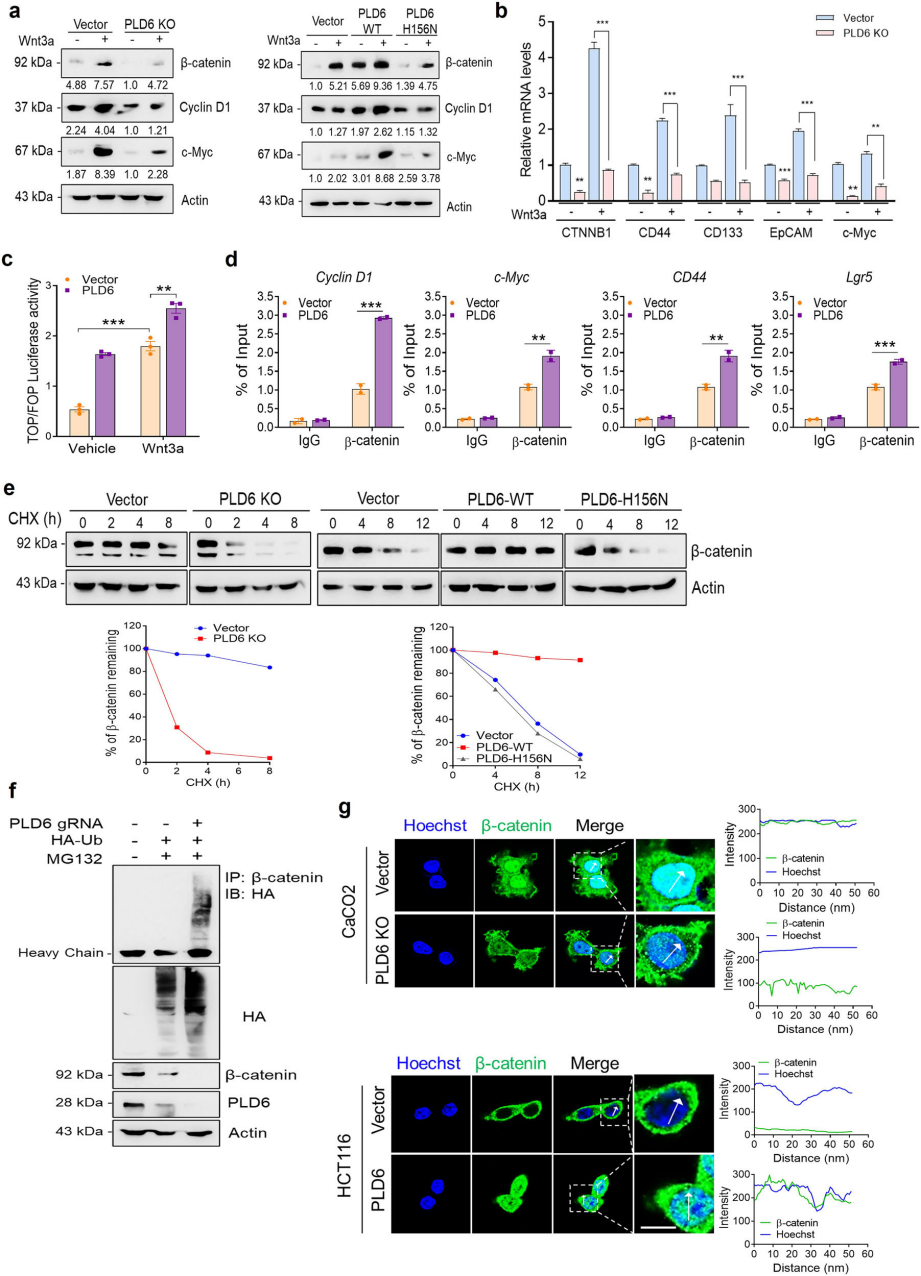
PLD6 upregulates Wnt/β-catenin signaling pathways. **a** The protein levels of β-catenin and its target genes were assessed in the indicated cells after treatment with recombinant Wnt3a (150 ng ml^−1^) for 6 h. **b** The expression levels of β-catenin and its target genes were analyzed in PLD6-KO Caco-2 cells using qRT–PCR. **c** The cells were cotransfected with the TOP/Flash or FOP/Flash reporter constructs and Renilla luciferase. After Wnt3a treatment for 4 h (or no treatment), the cell lysates were collected to measure luciferase activity. **d** A ChIP assay was performed using an anti-β-catenin antibody to demonstrate β-catenin binding to its target gene promoters. **e** The β-catenin stability was evaluated in Caco-2 and HCT116 cells treated with cycloheximide (CHX) (10 μg ml^−1^) for the indicated time points. **f** The HEK293T cells were treated with a single-guide RNA targeting PLD6 (sgPLD6) for 16 h to deplete PLD6, followed by treatment with or without MG132 (10 μM) for an additional 6 h. **g** The confocal microscopy images of PLD6-depleted Caco-2 cells and PLD6-overexpressing cells showing β-catenin localization (green) and nuclear colocalization (blue). The representative images were obtained from at least three fields. The results represent at least three independent experiments. Scale bar, 100 μm. Statistical significance: *P < 0.05, **P < 0.01, ***P < 0.001.

Considering the pivotal role of Wnt/β-catenin signaling in CRC, we investigated whether PLD6 regulates this pathway in CRC cells. In Caco-2 cells, PLD6 deficiency significantly downregulated the protein levels of Wnt3a-induced β-catenin and its target genes, including Cyclin D1 and c-Myc (Fig. 5a). Moreover, PLD6 KO reduced the mRNA levels of Wnt3a-induced β-catenin and its transcriptional target genes, including CD44, CD133, EpCAM and c-Myc (Fig. 5b). In contrast, overexpression of PLD6-WT—but not the H156N mutant—upregulated both basal and Wnt3a-induced β-catenin and its downstream target genes (Fig. 5a). Furthermore, elevated PLD6 expression significantly enhanced Wnt3a-induced transactivation, as demonstrated by the TOP/FOP flash assay, which assessed Wnt/β-catenin signaling activity (Fig. 5c). To explore the dynamic interaction between β-catenin and its target genes, we conducted ChIP assays, which revealed that PLD6 overexpression significantly increased the binding of β-catenin to cyclin D1, c-Myc, CD44 and LGR5 (Fig. 5d). We also assessed the effects of PLD6 on the β-catenin protein stability. PLD6 ablation accelerated the decline in β-catenin stability induced by cycloheximide treatment compared with that in the control cells (Fig. 5e). Conversely, overexpression of WT-PLD6 restored the stability of β-catenin, whereas overexpression of the H156N mutant did not (Fig. 5e). As β-catenin stability is regulated by the ubiquitin-mediated proteasomal pathway, our results showed that PLD6 KO promoted the ubiquitination and degradation of β-catenin (Fig. 5f). In addition, the enhanced stability of β-catenin owing to PLD6 overexpression led to its nuclear translocation in HCT116 cells, as observed by confocal microscopy (Fig. 5g). Conversely, PLD6 deficiency disrupted the nuclear localization of β-catenin (Fig. 5g). Overall, these findings indicate that PLD6 activates the Wnt signaling pathway by enhancing the stability and transcriptional activity of β-catenin and its target genes.

### PLD6-mediated acetyl-CoA promotes Wnt signaling through K49 acetylation of β-catenin

**Fig. 6:**
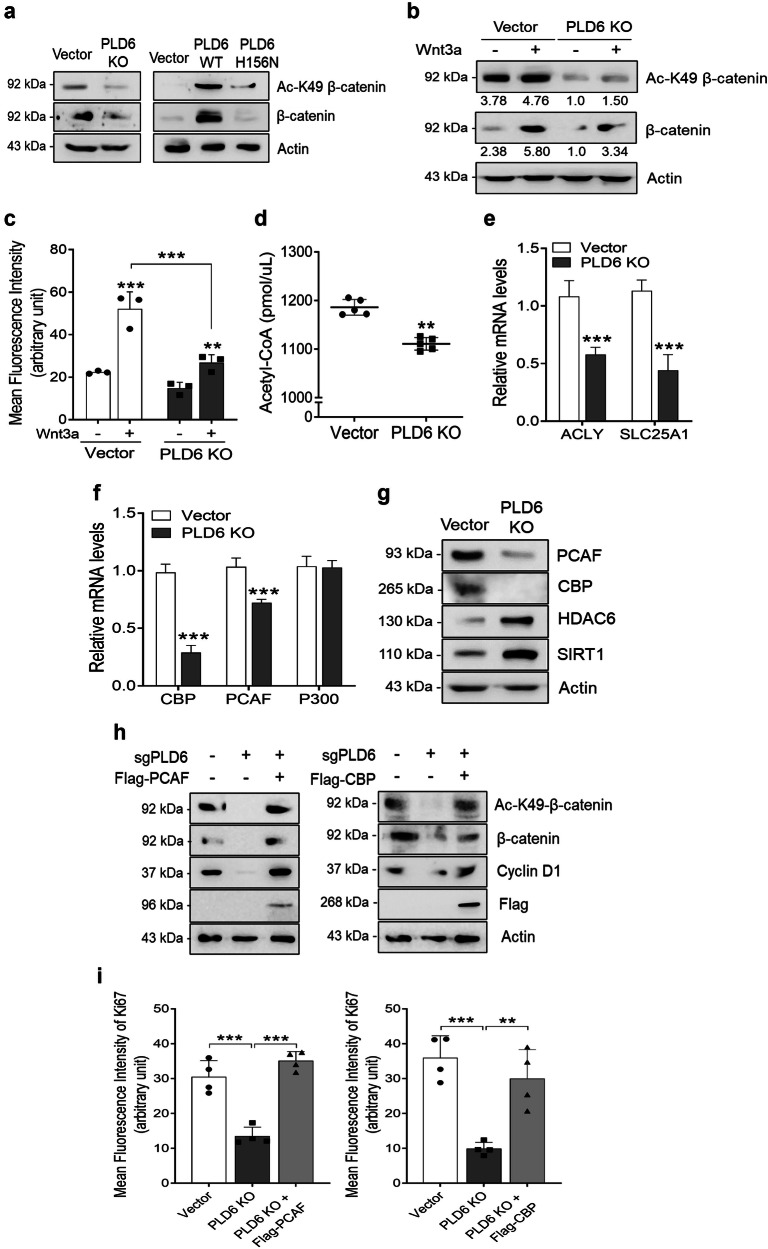
PLD6-mediated acetyl-CoA promotes Wnt signaling through K49 acetylation of β-catenin. **a** A western blot analysis of Ac-K49-β-catenin and total β-catenin levels in PLD6-deficient Caco-2 and PLD6-overexpressing HCT116 cells. **b**, **c** Ac-K49-β-catenin and total β-catenin levels in control and PLD6-KO Caco-2 cells were assessed following treatment with 150 ng ml^−1^ Wnt3a for 6 h, using immunoblotting (**b**) and immunofluorescence imaging (**c**). **d** Acetyl-CoA levels in control and PLD6-depleted Caco-2 cells were quantified using a fluorescence assay kit. **e**, **f** The mRNA levels of the indicated genes were measured using qRT–PCR. **g** The protein levels of key enzymes involved in the acetylation and deacetylation of β-catenin were evaluated in PLD6-KO cells using western blotting. **h**, **i** The HEK293T cells were treated with sgPLD6 for 16 h and subsequently transfected with either Flag-CBP or Flag-PCAF for an additional 16 h; a western blot analysis (**h**) and confocal microscopy imaging with Ki67 (**i**) were performed to assess protein expression and proliferation. The results represent at least three independent experiments. Statistical significance: *P < 0.05, **P < 0.01, ***P < 0.001.

Acetylation of β-catenin is known to significantly enhance its stability, nuclear translocation and transcriptional activity^35^. In addition, Wnt signaling influences mitochondrial function by altering mitochondrial dynamics and metabolism^36,37^. β-catenin is known to be acetylated by various lysine acetyltransferases, including CREB-binding protein (CBP)^38^, P300 (ref. ^39^) and PCAF^40^. P300 is responsible for acetylating K345 of β-catenin, whereas CBP acetylates K49. PCAF can acetylate both K19 and K49 of β-catenin, enhancing its stability and nuclear translocation, as well as increasing its affinity for T cell factor/lymphoid enhancer factor transcription factors. As PLD6 increases the stability and transcriptional activity of β-catenin, we explored whether PLD6 affects β-catenin acetylation. Ac-K49-β-catenin is critical for the transcriptional activation of Wnt target genes; therefore, we examined the impact of PLD6 on Ac-K49-β-catenin levels. Notably, PLD6 depletion downregulated both Ac-K49-β-catenin and total β-catenin levels, whereas overexpression of PLD6-WT but not PLD6-H156N increased the Ac-K49-β-catenin and β-catenin levels (Fig. 6a). Moreover, Wnt3a-induced Ac-K49-β-catenin and β-catenin levels were reduced in PLD6-deficient Caco-2 cells, as shown by western blot and immunofluorescence analyses (Fig. 6b, c and Supplementary Fig. 4a). Considering that PLD6 enhances mitochondrial metabolism, as shown in Figs. 3 and 4, we hypothesized that PLD6 may regulate β-catenin acetylation through TCA cycle metabolites, particularly acetyl-CoA. Citrate, derived from the TCA cycle, can be exported to the cytosol, where it is converted to acetyl-CoA, which then participates in the acetylation of target proteins. We observed a substantial decrease in the acetyl-CoA levels in PLD6-KO cells (Fig. 6d). Furthermore, PLD6 deficiency significantly reduced the expression of SLC25A1 (a mitochondrial citrate transporter) and ATP citrate lyase (ACLY) (Fig. 6e). PLD6 KO also suppressed the expression of CBP and PCAF (Fig. 6f), suggesting that PLD6-driven acetyl-CoA contributes to β-catenin stabilization through acetylation. Notably, PLD6 deficiency upregulated the expression of lysine deacetylases for β-catenin, including HDAC6 and SIRT1 (ref. ^35^) (Fig. 6g), implying that both lysine deacetylases and acetyltransferases such as PCAF and CBP are involved in PLD6-mediated K49 acetylation of β-catenin. We then confirmed that ectopic expression of PCAF or CBP in PLD6-KO cells rescued β-catenin acetylation and the expression of its transcriptional target gene, cyclin D1 (Fig. 6h). Finally, we demonstrated that the proliferation of Caco-2 cells, which was suppressed by PLD6 ablation, was restored by the ectopic expression of CBP and PCAF (Fig. 6i and Supplementary Fig. 4b). These results suggest that PLD6-mediated β-catenin acetylation plays a role in CRC cell proliferation. Further, PLD6-mediated acetyl-CoA induces β-catenin acetylation with the assistance of CBP and PCAF, promoting CRC cell proliferation through the activation of Wnt signaling.

### Activation of Wnt/β-catenin signaling by PLD6 induces stem-like traits and chemoresistance in CRC cells

**Fig. 7:**
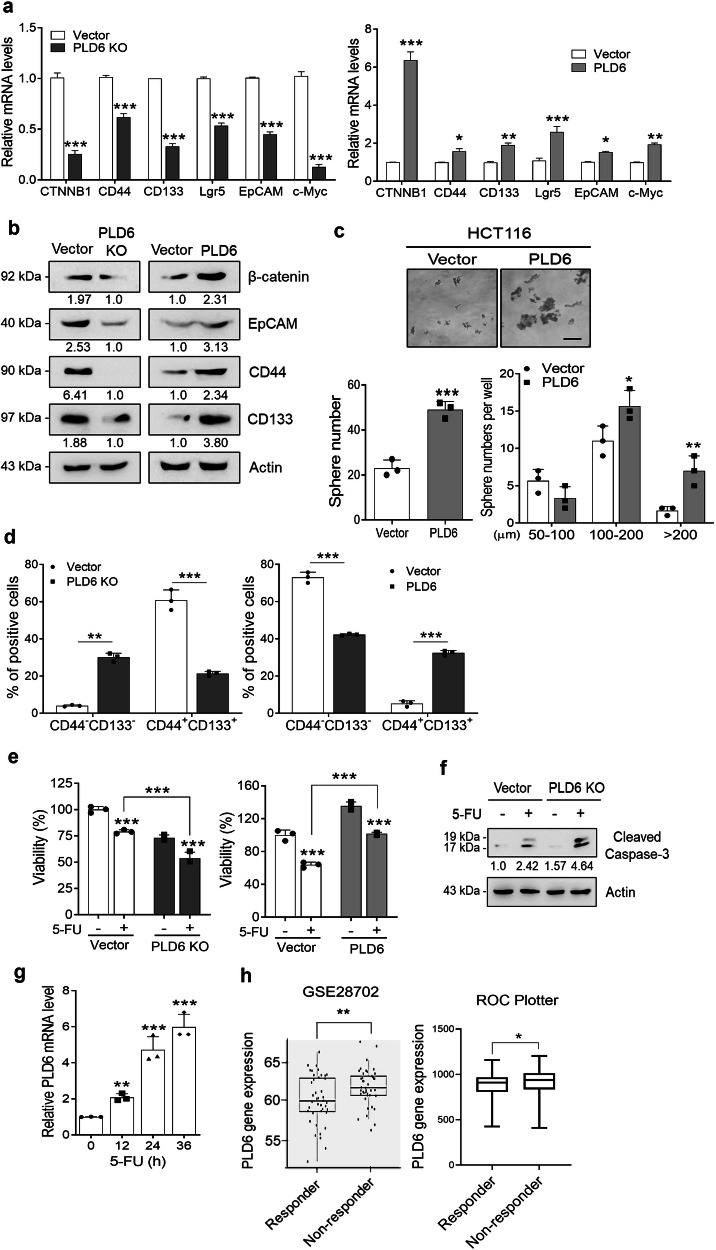
Activation of Wnt/β-catenin signaling by PLD6 induces stem-like traits and chemoresistance in CRC cells. **a** The mRNA expression levels of β-catenin (CTNNB1) and downstream cancer stem cell (CSC)-related genes were analyzed in PLD6-KO and PLD6-overexpressing cell lines using qRT–PCR. **b** A western blot analysis was performed to validate the protein expression of CSC-related genes. **c** The sphere-forming ability of HCT116 cells was assessed following PLD6 modulation. **d** A flow cytometric analysis of CD44^+^CD133^+^ subpopulations was conducted in PLD6-KO Caco-2 cells and PLD6-overexpressing HCT116 cells, with results presented as percentages of double-negative and double-positive populations. **e** PLD6-KO and overexpressing cell lines were treated with 10 μM of 5-FU for 48 h, and the cell viability was evaluated using the WST-1 assay. **f** 5-FU-induced apoptosis was assessed by detecting cleaved caspase-3 using immunoblotting in both cell lines. **g** PLD6 mRNA expression was measured in Caco-2 cells treated with 5-FU at the indicated time points. **h** The PLD6 expression levels were compared between nonresponder and responder groups in colon cancer cohorts using data from GSE28702 and the ROC plotter server. The results represent at least three independent experiments. Scale bar, 100 μm. Statistical significance: *P < 0.05, **P < 0.01, ***P < 0.001.

Activation of Wnt/β-catenin signaling is known to regulate cancer stem cells or cancer-initiating cells. Target genes of this pathway, including CD44, CD133, Lgr5, EpCAM and c-Myc, are frequently used as markers of colon cancer-initiating cells. Therefore, we investigated whether PLD6 regulates the expression of Wnt/β-catenin target genes, which are involved in CRC cell stemness. PLD6-deficient Caco-2 cells exhibited downregulation of stemness-associated markers, whereas overexpression of PLD6 in HCT116 cells significantly increased their expression (Fig. 7a, b). To further explore the role of PLD6 in the self-renewal capability of CRC cells, we performed a sphere formation assay. PLD6 overexpression significantly increased the average diameter and number of spheres formed by the HCT116 cells (Fig. 7c). Further, PLD6 deficiency substantially reduced the population of CD44^+^CD133^+^ cells compared with that of the control cells. Conversely, PLD6 overexpression significantly increased the CD44^+^CD133^+^ population and decreased the CD44^–^CD133^–^ population (Fig. 7d and Supplementary Fig. 5a). These results suggest that PLD6 plays a critical role in the maintenance of colon cancer-initiating cells. As the maintenance and expansion of cancer stem cells within the tumor microenvironment often confers chemoresistance, we next examined whether PLD6 affects the sensitivity of cancer cells to 5-fluorouracil (5-FU), a chemotherapeutic agent commonly used in CRC treatment. As shown in Fig. 7e, f, PLD6 KO sensitized the cells to 5-FU-induced apoptosis and accelerated the decrease in 5-FU-induced cell viability. In contrast, PLD6 overexpression protected cells from 5-FU-induced apoptosis and preserved cell viability compared with that of control cells. Interestingly, 5-FU treatment upregulated PLD6 expression in Caco-2 cells (Fig. 7g). Finally, we used data from the GEO dataset and ROC plotter to show that PLD6 was overexpressed in patients with CRC showing no response to 5-FU^41^ (Fig. 7h). Collectively, these findings suggest that PLD6 promotes stem-like traits and chemoresistance in CRC cells, potentially through the activation of Wnt/β-catenin signaling.

### Targeting PLD6 attenuates tumorigenesis of CRC in vivo

**Fig. 8:**
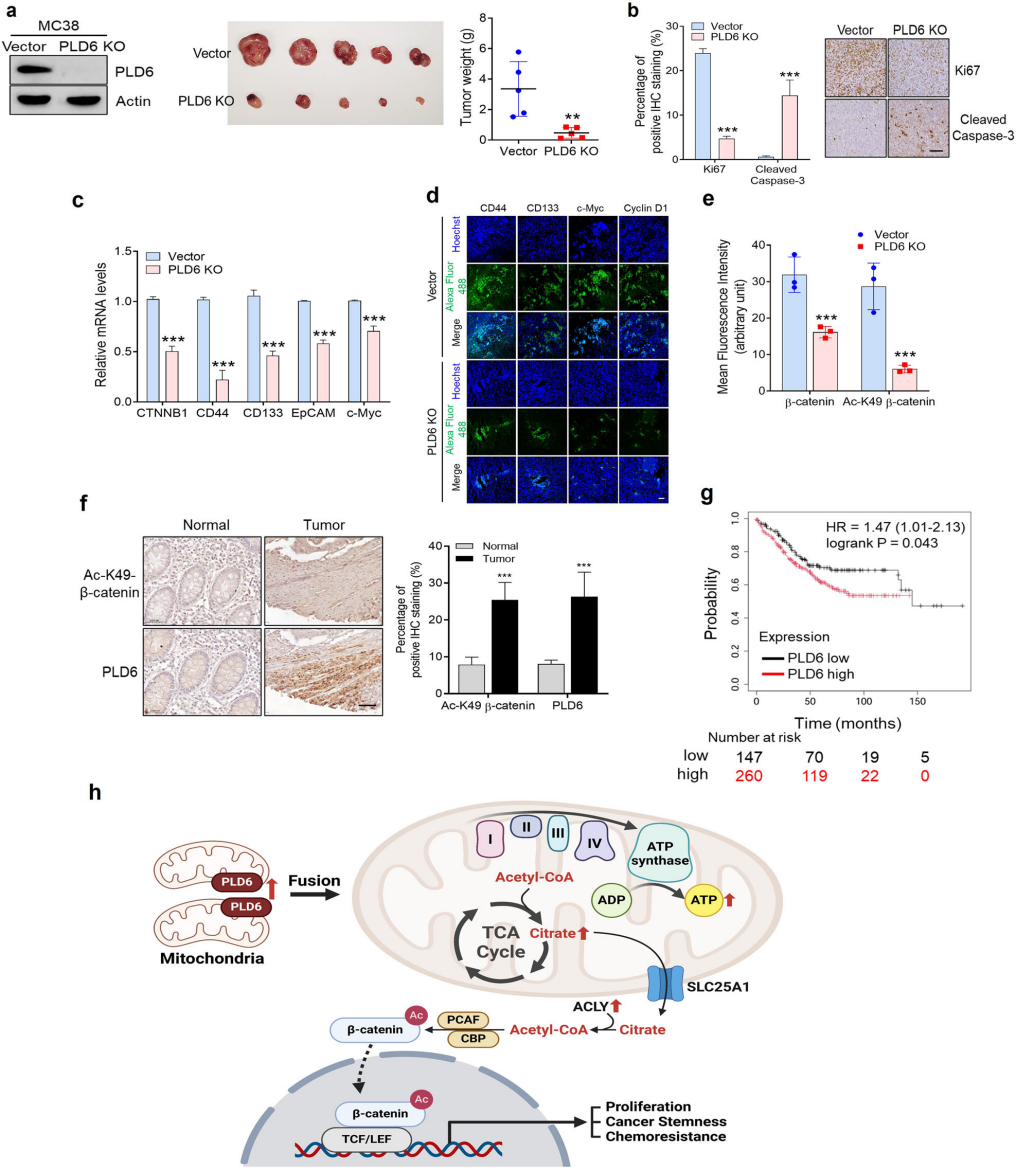
Targeting PLD6 attenuates CRC tumorigenesis in vivo. **a** The orthotopic tumor model: 1 × 10^6^ PLD6-depleted MC38 cells or vector control cells were injected into the cecal submucosa of C57BL/6 mice. The representative images of tumors were captured at 21 days post injection, and the tumor weights were measured. **b** The tumor tissues were sectioned, deparaffinized and stained with antibodies against Ki67 (a proliferation marker) and cleaved caspase-3 (an apoptosis marker). **c** qPCR was performed to detect β-catenin and the β-catenin target genes in tumor tissues. **d**, **e** The β-catenin target gene expression and β-catenin acetylation were evaluated using immunofluorescence (IF). **f** An IHC analysis was performed on a CRC TMA to assess the parallel expression of PLD6 and Ac-K49 β-catenin, and the percentage of positive IHC staining for these proteins was quantified. **g** The impact of PLD6 expression on overall survival in patients with CRC showing high β-catenin expression was analyzed using the Kaplan–Meier Plotter online database. **h** A working model explaining how the PLD6 promotes tumorigenesis in CRC via mitochondrial metabolism and Wnt signaling. The representative images were obtained from at least three fields. The results represent at least three independent experiments. Scale bar, 100 μm. Statistical significance: *P < 0.05, **P < 0.01, ***P < 0.001.

To investigate whether PLD6 regulates CRC tumorigenesis in vivo, we used an orthotopic tumor model in which PLD6-deficient MC38 cells were injected into the cecal submucosa of mice. The PLD6-KO cells were generated using the CRISPR–Cas9 system (Fig. 8a). PLD6-deficient cells showed significantly lower tumor size, weight and volume compared with those of control cells (Fig. 8a). The tumor tissues derived from mice injected with PLD6-KO cells exhibited reduced proliferation and increased apoptosis compared with those from control cell-injected mice, as indicated by IHC staining for Ki67 and active caspase-3 (Fig. 8b). In addition, tumors from PLD6-KO cells showed decreased expression of mesenchymal markers, such as N-cadherin, fibronectin and vimentin, and increased expression of epithelial markers, including E-cadherin, claudin-5, ZO-1 and occludin, as demonstrated using IHC (Supplementary Fig. 6a). PLD6 depletion also led to reduced expression of β-catenin and β-catenin transcriptional targets, including CD44, CD133, EpCAM and YAP, as analyzed using qPCR and immunofluorescence (Fig. 8c, d). PLD6 KO also significantly decreased the overall levels and nuclear accumulation of β-catenin and Ac-K49-β-catenin, as analyzed using immunofluorescence (Fig. 8e and Supplementary Fig. 6b). Consistent with the orthotopic model, PLD6-KO cells showed significantly attenuated tumor development in the subcutaneous model (Supplementary Fig. 6c, d). PLD6 KO suppressed CRC cell proliferation and increased apoptosis in tumor tissues, as indicated by IHC analysis (Supplementary Fig. 6e). Furthermore, epithelial markers were upregulated, whereas mesenchymal markers were downregulated in PLD6-KO tumors, consistent with the subcutaneous model (Supplementary Fig. 6f). Further, PLD6-deficient tumors exhibited reduced expression of β-catenin transcriptional targets and Ac-K49-β-catenin, as analyzed using IHC (Supplementary Fig. 6g). To assess the physiological relevance, we evaluated the parallel expression between PLD6 and Ac-K49-β-catenin in CRC tissues from human patients. An IHC staining was performed on TMAs, which included 24 tumor tissues and their corresponding nontumor adjacent tissues to analyze the expression levels of each protein. The IHC analysis revealed that the expression levels of PLD6 and Ac-K49-β-catenin were higher in CRC tissues compared with normal colon tissues (Fig. 8f). Consistent with our previous in vitro findings, a positive correlation between PLD6 and acetylated β-catenin was observed in the in vivo specimens. Overall, these results indicated that PLD6 ablation suppressed CRC tumorigenesis in vivo, possibly through the inhibition of Wnt signaling. We then conducted a survival analysis using Kaplan–Meier plots to evaluate the prognostic impact of PLD6 in the context of the Wnt signaling pathway. Among patients with high β-catenin expression, those with elevated PLD6 levels demonstrated significantly poor overall survival. These findings highlight PLD6 as a critical marker for identifying high-risk patients within β-catenin-high groups (Fig. 8g). Collectively, these results indicate that targeting PLD6 attenuates CRC tumorigenesis in vivo.

## Discussion

The cancer cells are characterized by rapid cell division and high demand for energy and macromolecules. To meet these needs, the cells undergo metabolic reprogramming, which results in distinct metabolic features. In this study, we reveal a novel role of PLD6 in CRC, specifically in regulating mitochondrial metabolism. We identified PLD6-mediated mitochondrial metabolism as a key regulator of Wnt signaling and tumor progression in CRC. The mitochondria are dynamic organelles regulated by fission and fusion processes. PLD6 has been reported to induce mitochondrial fusion via generation of phosphatidic acid on the mitochondrial outer membrane. In addition, PLD6 plays important roles in spermatogenesis via piRNA biogenesis^5,7^ and steroidogenesis^42^ in mitochondria. Notably, piRNAs and piRNA pathway genes are increasingly being recognized for their roles in cancer development and progression^43,44^. Moreover, mitochondrial dynamics are altered during oncogenic transformation^45^. Furthermore, the canonical PLD isoforms (PLD1 and PLD2) are well known to be implicated in cancer by activating oncogenic pathways. However, the role of PLD6 in cancer has not yet been elucidated. Our study demonstrates that PLD6 is upregulated in CRC and is associated with poor prognosis. PLD6 overexpression enhances the oncogenic potential of CRC cells and regulates mitochondrial biomass, calcium levels, ROS levels and membrane potential, all of which are critical for cellular processes. Therefore, it is suggested that the oncogenic potential of PLD6 is linked to the dysregulation of mitochondrial functions, including metabolism. We demonstrate that the catalytic activity of PLD6 is critical for mitochondrial metabolic function. The possibility that the canonical PLDs also affect mitochondrial function via phosphatidic acid cannot be excluded. Notably, the inhibitors of mitochondrial respiration, such as metformin, suppress PLD6-mediated proliferation of CRC cells, suggesting that PLD6 promotes oncogenesis through mitochondrial metabolism. Further studies are needed to determine whether metformin could be one of strategies to inhibit PLD6-mediated tumorigenesis. Our findings support a model in which PLD6-dependent mitochondrial activity is essential for maintaining Wnt/β-catenin signaling in CRC cells. PLD6 upregulates β-catenin at both the protein and transcriptional levels. At the protein level, PLD6 enhances β-catenin stability by promoting its acetylation, which in turn increases its nuclear translocation and transcriptional activity. Moreover, PLD6 also increases β-catenin mRNA expression. Previously, pharmacological inhibition of PLD1 was shown to upregulate miR-320a and miR-4496 in CRC, both of which bind to the 3′ untranslated region of β-catenin and downregulate its expression post-transcriptionally^28,46^. Moreover, miR-320a and miR-4496 suppress gastric tumorigenesis and metastasis by targeting β-catenin^46^. Therefore, the possibility that PLD6-induced β-catenin upregulation is linked to the downregulation of miR-320a and miR-4496 warrants further investigation. We identified acetyl-CoA as a key metabolite mediating the crosstalk between PLD6 and Wnt/β-catenin signaling. PLD6 increases the levels of citrate, a TCA cycle metabolite which is then converted to acetyl-CoA by ACLY in the cytosol. ACLY has been reported to acetylate β-catenin at K49 in hepatocarcinoma cells^47^. This acetylation improves β-catenin stability by inhibiting its ubiquitination and promotes its nuclear translocation, thus enhancing Wnt-dependent gene transcription^39^. PLD6 activity is responsible for CBP/PCAF-induced β-catenin acetylation at K49 via increased acetyl-CoA production. In patients with CRC who do not respond to 5-FU treatment, as well as in 5-FU-treated CRC cells, increased PLD6 expression is likely associated with chemoresistance through the activation of Wnt/β-catenin signaling. Ultimately, PLD6-induced acetylation of β-catenin is linked to the proliferation and stemness of CRC cells. Targeting PLD6 in both subcutaneous and orthotopic cancer mouse models attenuated tumorigenesis possibly by suppressing Wnt/β-catenin signaling, thereby sensitizing CRC cells to 5-FU-induced apoptosis. Our study highlights the functional significance of PLD6-mediated mitochondrial metabolism in promoting tumorigenesis via activation of Wnt signaling in CRC. By establishing the role of mitochondrial retrograde signaling in regulating cancer stem cell traits and chemoresistance, we demonstrated that targeting mitochondrial metabolism may be a promising therapeutic strategy for CRC (Fig. 8h). Thus, future studies identifying and developing effective PLD6 inhibitors are crucial for overcoming CRC malignancy and recurrence.

## Supplementary information

Supplementary Information
