# Supplementary material for: Phospholipase D6 activates Wnt/β-catenin signaling through mitochondrial metabolic reprogramming to promote tumorigenesis in colorectal cancer
Source: Exp Mol Med. 2025 Apr 21;57(4):910–24. doi: 10.1038/s12276-025-01446-9 (PMC12046002; doi:10.1038/s12276-025-01446-9)
Supplement: Supplementary file 1 — Supplementary Information [file 12276_2025_1446_MOESM1_ESM.pdf]

Supplementary Information

Supplementary figures

Supplementary Fig.1 PLD6 promotes the tumorigenic potential of CRC cells.

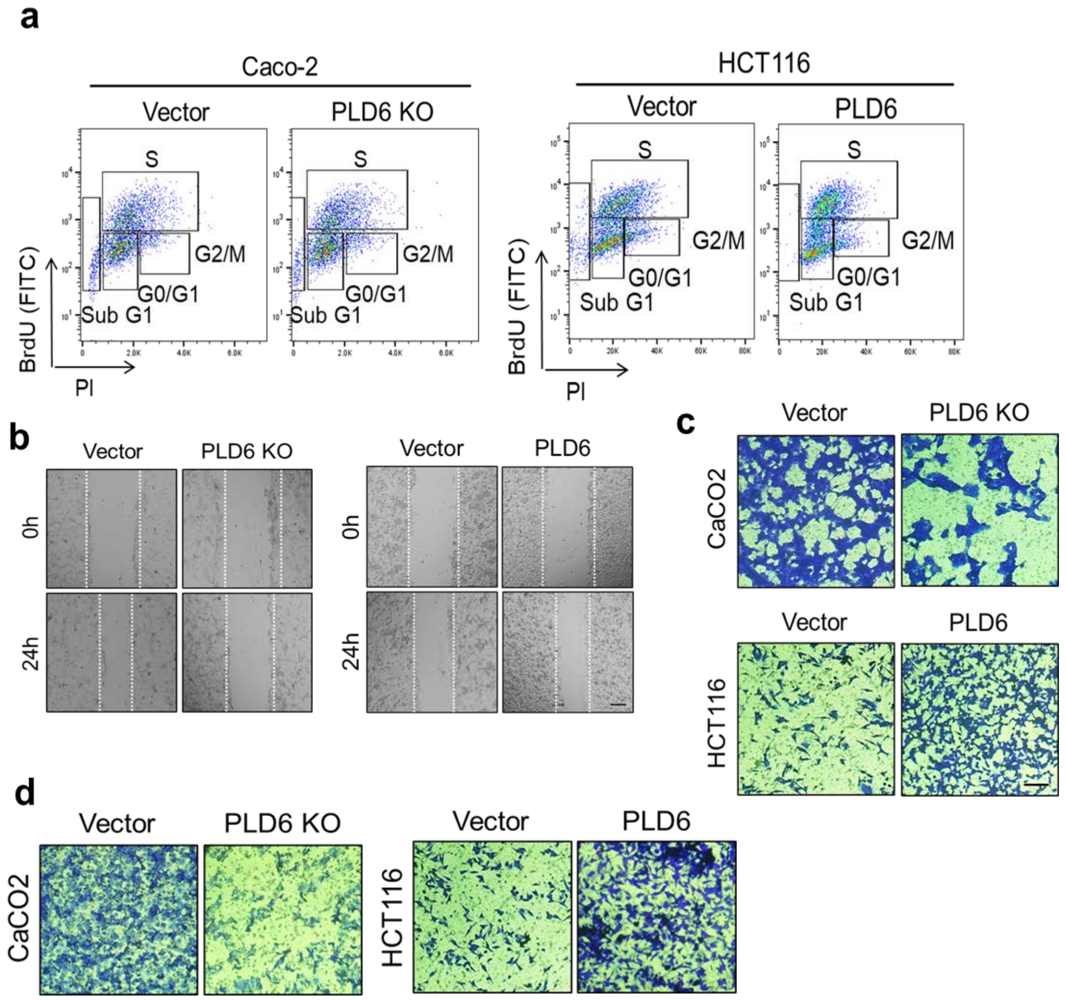


(a) The effect of PLD6 on cell cycle was assessed using BrdU and PI in CRC cells. (b, c) The effect of PLD6 on cell migration was evaluated through wound healing and transwell migration assay. (d) Invasion was quantified via transwell chamber coated with Matrigel. Representative images were obtained from at least three fields. Scale bar: 100 μm

Supplementary Fig.2 PLD6 enhances mitochondrial membrane potential, metabolism and OXPHOS

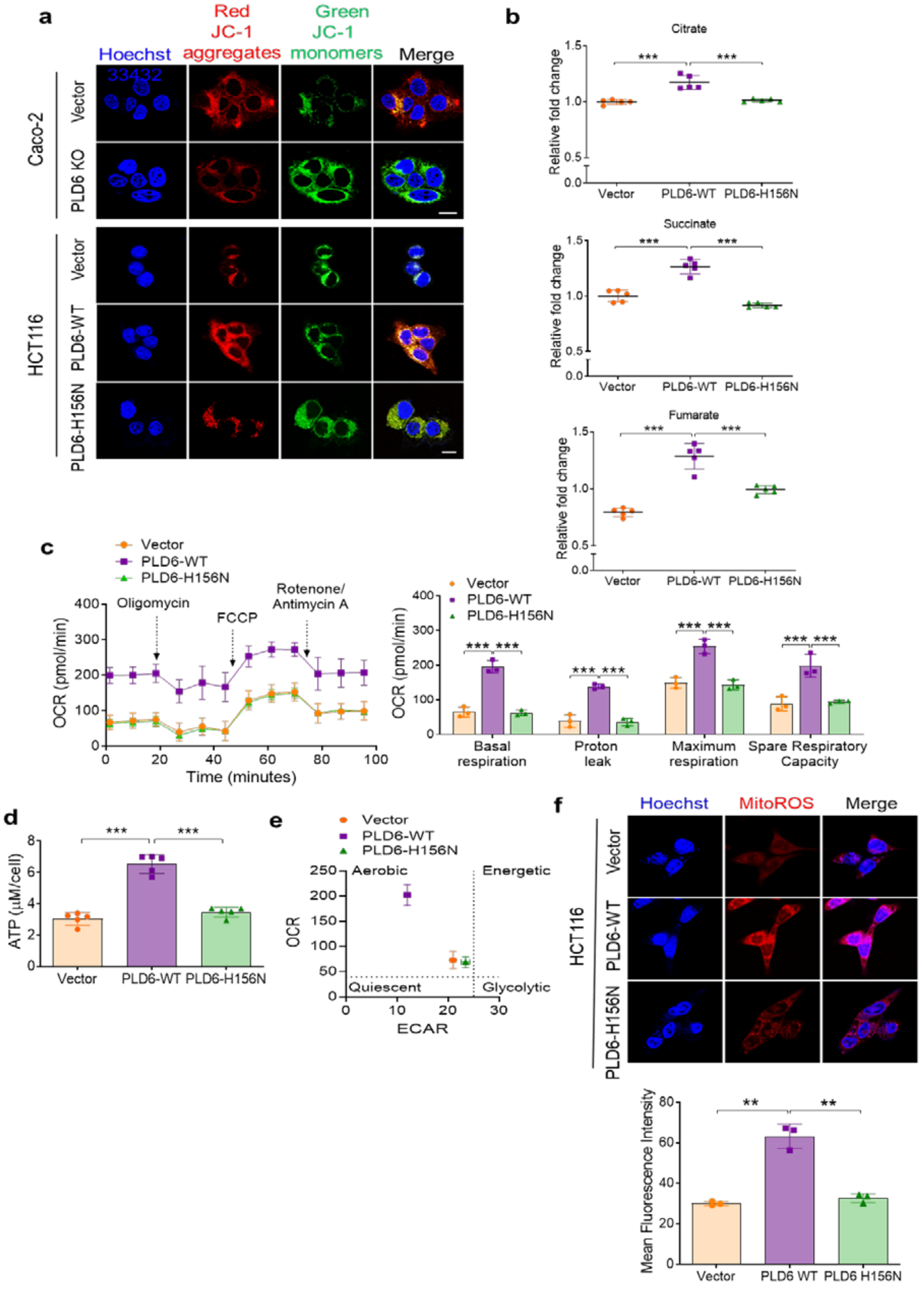

(a) The effect of PLD6 on mitochondrial membrane potential was assessed using JC-1 dye in CRC cells. (b) The impact of PLD6 on the generation of TCA cycle metabolites was measured in CRC cells. (c) OCR was evaluated through Seahorse Extracellular Flux Analyzer. (d) The effect of PLD6 on the production of ATP was measured in CRC cells. (e) Scatter plot of OCR and ECAR analysis. (f) Mitochondrial ROS levels were visualized and quantified in CRC cells. Representative images were obtained from at least three fields. Scale bar: 100 μm. *Statistical significance: *$p<0.05$, **$p<0.01$, ***$p<0.001$.

Supplementary Fig.3 PLD6 facilitates oncogenic progression in CRC cells through the upregulation of mitochondrial metabolism

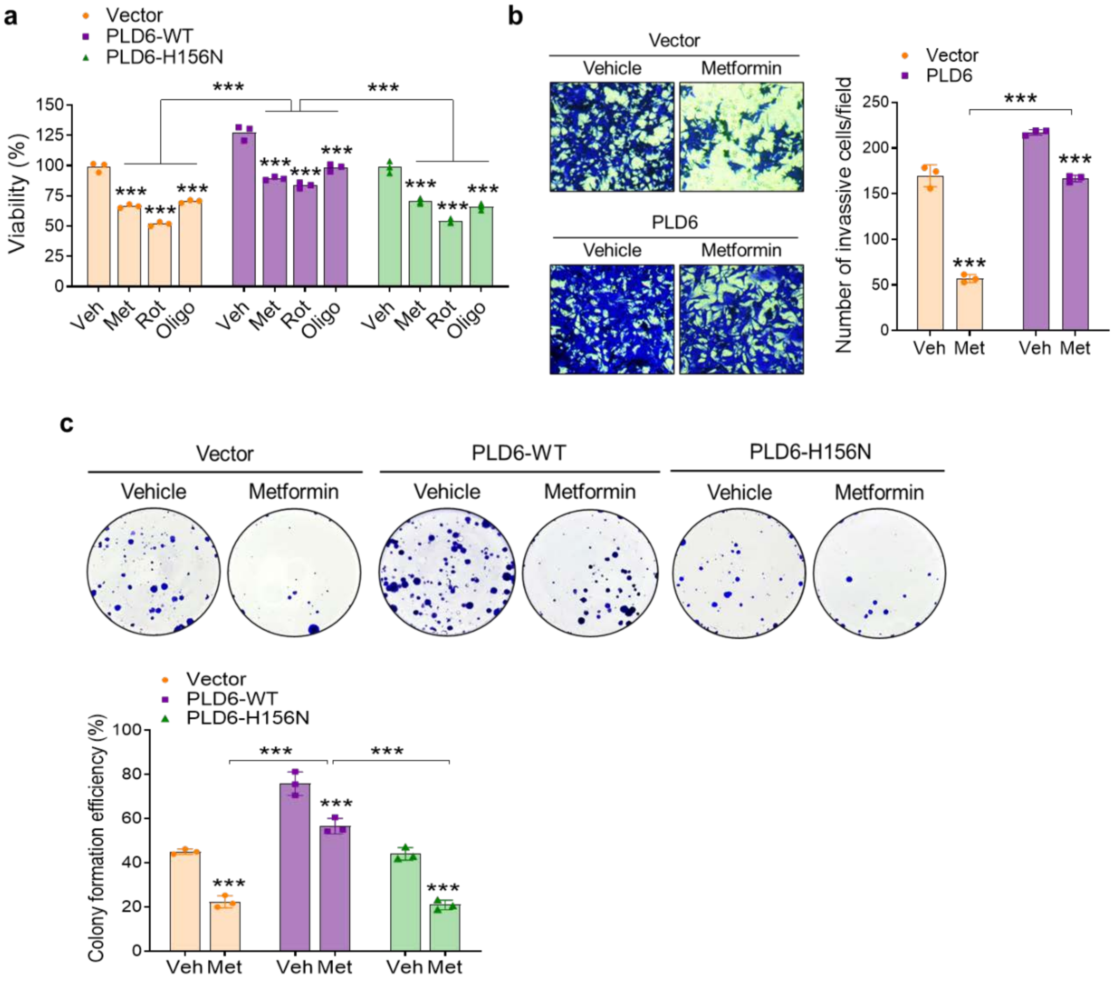

(a) Cell viability was assessed using the WST-1 assay in CRC cells following treatment with the Inhibitors of mitochondrial respiration. (b) The effect of PLD6 on Invasive capacity of CRC cells was evaluated in response to Metformin treatment. (c) Colony formation was assessed in CRC cells following treatment with Metformin. Results represent at least three independent experiments. Scale bar: 100 μm. *Statistical significance: *p<0.05, **p<0.01, ***p<0.001.

Supplementary Fig.4 PLD6 promotes K49 acetylation of β-catenin through PCAF and CBP

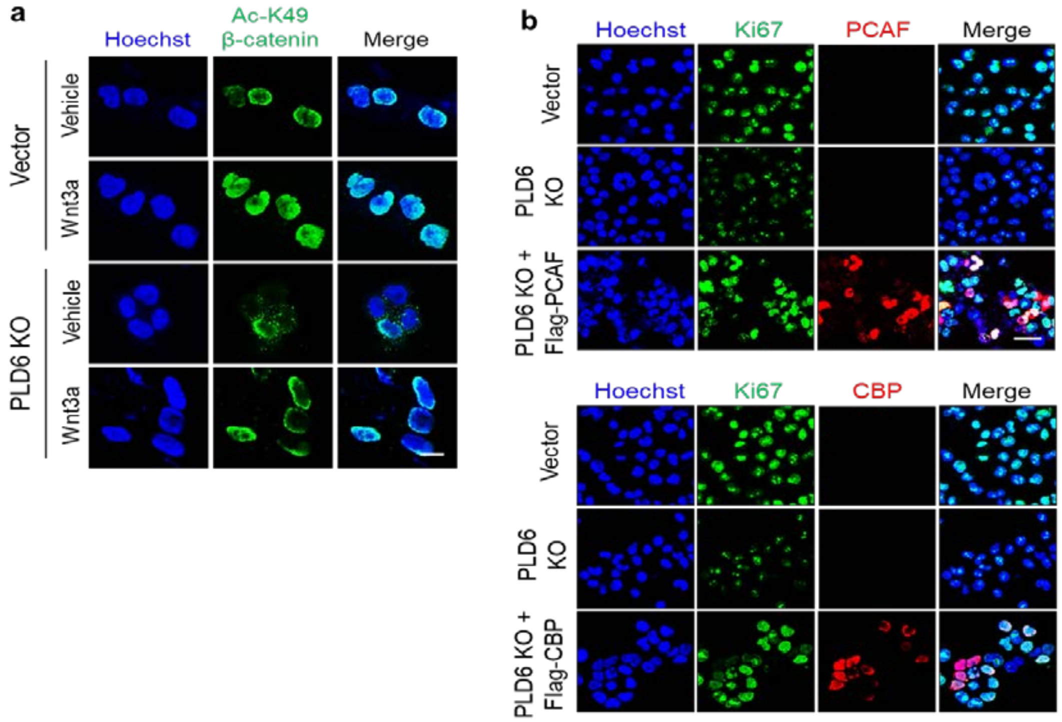


(a)The effect of PLD6 on Ac-K49-β-catenin levels was assessed using Immunofluorescence in CRC cells, with or without Wnt3a. (b) Cell proliferation was measured by staining with Ki67. Representative images were obtained from at least three fields. Scale bar: 100 μm.

Supplementary Fig.5 PLD6 enhances stem cell-like characteristics in CRC cells

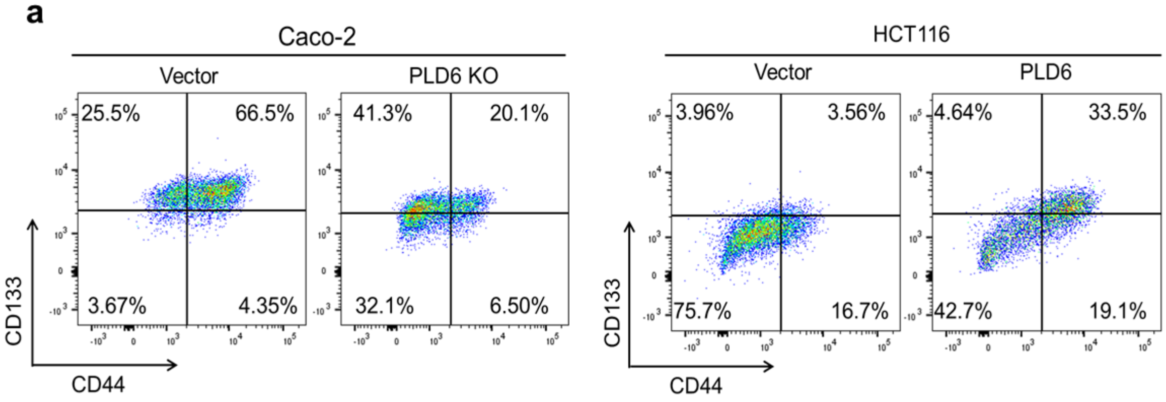


(a) CD44$^+$CD133$^+$ population was evaluated using FACS in CRC cells. Results represent at least three independent experiments

Supplementary Fig.6 PLD6 deficiency suppresses colorectal cancer progression In vivo

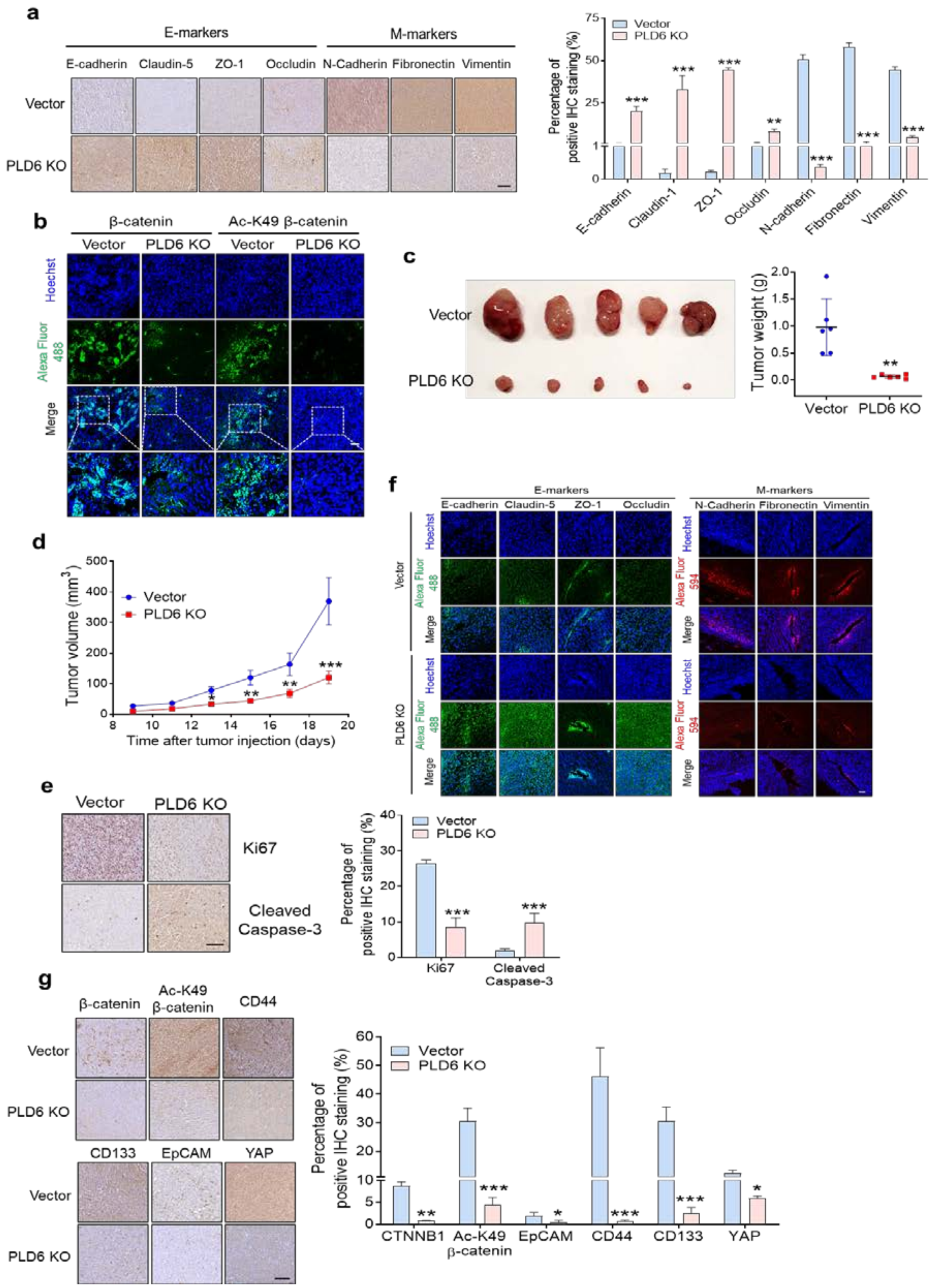

(a) Immunohistochemistry (IHC) analysis of EMT associated proteins in orthotopic models. (b) Immunofluorescence (IF) analysis of β-catenin and Ac-K49-β-catenin in orthotopic models. (c) Subcutaneous tumor model: 2 × $10^5$ PLD6-depleted MC38 cells or vector control cells were subcutaneously injected into C57BL/6 mice. Representative images of tumors were captured at 21 days post-injection, and tumor weights were measured. (d) Tumor volumes were recorded every two days to monitor growth kinetics. (e) IHC and IF analysis of Ki67, cleaved caspase-3, (f) EMT associated proteins (g) Wnt signaling proteins in subcutaneous models. Representative images were obtained from at least three fields. Scale bar: 100 μm.

Supplementary Table

Supplementary Table 1. gRNA sequences targeting PLD6

| Target | Oligonucleotide Sequences (5’ - 3') |
|---|---|
| hPLD6 g1 | cacc GCA GTT GCT GCA CCA GCG TG |
| mPLD6 g1 | cacc GGG TCT CGC GCT GGC CCT AG |

Supplementary Table 2. Primer sequences used in qRT-PCR analysis

| Genes | Dir. | Primers (5'-3') | Genes | Dir. | Primers (5'-3') |
|---|---|---|---|---|---|
| SUCLG1 | F | TAT GGC ACC AAA CTC GTT GGA | NDUFA2 | F | CAT TCA CTT ATG CCA GCG TTC CCG |
| | R | GAA GCC GTT GCT CCT GTC T | | R | GGA TTA GAA TGG GCA GGT |
| FH | F | GGA GGT GTG ACA GAA CGC AT | NDUFA11 | F | AAG GAG TGG CTA AGG TGG AC |
| | R | CAT CTG CTG CCT TCA TTA TTG C | | R | CAC CGA GGA AGT AGT TCA GG |
| MDH2 | F | TCG GCC CAG AAC AAT GCT AAA | SDHB | F | ATC TTG TTC CCG ATT TGA GC |
| | R | GCG GCT TTG GTC TCG ATG T | | R | GTC TCC GTT CCA CCA GTA CG |
| ACYL | F | CCC CAA GAT TCA GTC CCA AGT | SDHD | F | ATG GCG GTT CTC TGG AGG CTG |
| | R | GCC TTG GTA TGT CGG CTG AA | | R | GAG CTT CCA CAG CAT GGC AAC |
| CBP | F | TGA GGA TCT CAT GGT AAA CAG C | UQCRB | F | ATG GCT GGT AAG CAG GCC |
| | R | AAG AAT ATG GCT CCG ATT GC | | R | CTT CTT TGC CCA TTC TTC |
| SLC25A1 | F | CCC CAT GGA GAC CAT CAA G | CYC-1 | F | GCA TGG TGG TGA GGA CTA CG |
| | R | CCT GGT ACG TCC CCT TCA G | | R | GGC CAG GAA AGT AGG GGT TG |
| P300 | F | CCC TGG AGG CAT TAT AGG AGA | COX5B | F | CCC AAA GGG AGC TTC AGG |
| | R | AAG CAC CAG TGT CTC AAG CA | | R | CGA CGC TGG TAT TGT CCT CT |
| PCAF | F | GCT TTA CAG CAC GCT CAA GA | COX6A1 | F | CAT CAG GAC CAA GCC GTT TC |
| | R | GTT CCA TGA AGG GCC AAG | | R | TTT CTG GTC CAT GTG CAG AGT AAC |
| PLD6 | F | AGC TCC CCG AGG GCT C | ATP5D | F | CAAGATGCCTTTGAGTGTTCAA |
| | R | CAG AGA TCC AGG CTG GCG | | R | GGTCTTCTTTTCCCAAGACAAC |
| CTNNB1 | F | GCT TGC CTT GCT CAA CAA AA | ATP5J | F | TCAGCCGTCTCAGTCCATTT |
| | R | TCC CAA GGA GAC CTT CCA TC | | R | CCAAACATTTGCTTGAGCTT |
| CD44 | F | AAG ACA TCT ACCCCA GCA AC | PDH | F | AGG CTG GCA TAA ACC CTA CG |
| | R | CCA AGA TGA TCA GCC ATT CTG G | | R | GCC GTT GCC TCC ATA GAA GT |
| CD133 | F | TCA GGA TTT TGC TGC TTG TG | CS | F | TGC TTC CTC CAC GAA TTT GAA A |
| | R | GCA GTA TCT AGA GCGGTG GC | | R | CCA CCA TAC ATC ATG TCC ACA G |
| LGR5 | F | CCT GGA GTT ACG TCT TGC G | ACO2 | F | CCC TAC AGC CTA CTG GTG ACT |
| | R | CTG GAG AGG TTT CCA AAG GC | | R | TGT ACT CGT TGG GCT CAA AGT |
| EpCAM | F | GGC OGT AAA CTG CTT TGT GA | IDH1 | F | TGC TGA GTT TGC CTT TGA GTA TG |
| | R | TTG AGC CAT TCA TTT CTG CC | | R | CGC ATG ATG TTG GCT TTG TG |
| c-myc | F | CAC CAG CAG CGA CTC TGA | IHD3 | F | GAG CCA AGT CTC AGC GGA TT |
| | R | GAT CCA GAC TCT GAC CTT TTG C | | R | GGG CAT CAC AAG CAC ATC AAA |

Supplementary Table 3. Primer sequences for ChIP assay

| Genes | Dir. | Primers (5'-3') |
|---|---|---|
| CD44 | F | ACA CTC CAG GTT CCC CGA CCC |
| | R | GAA GGA CAC ACC CAA GCA AGG |
| Cyclin D1 | F | GCT TTC CAT TCA GAG GTG TG |
| | R | CCG AAA ATT CCA GCA GCA GC |
| c-myc | F | GAG GGA CCA AGG ATG AGA AGA ATG T |
| | R | CCC CAC ACA TGA TTT GTT TGC TCC |
| LGR5 | F | GGG GCA ACT TGT GAC GTT AGG A |
| | R | GCC TGT TTT CTT GGG TTG CTG TAA AC |

Supplementary Table 4**.** Association between the clinicopathological characteristics and PLD6 expression in Colorectal Cancer

| Stage | No. of samples | No. of positive samples (%) | P-value |
|---|---|---|---|
| 1 | 52 | 23 (44.2%) | <0.001 |
| 2 | 76 | 38 (50%) | |
| 3 | 4 | 3 (75%) | |
| 4 | 4 | 4 (100%) | |
